# Dr. Charles T. Parkes

**Published:** 1891-05

**Authors:** 


### Obituary.

---

DR. CHARLES T. PARKES, Professor of Surgery in Rush Medical College, Chicago, Ill., died after a brief illness on Saturday, March 28, 1891, of pneumonia. Dr. Parkes stood conspicuously in the front rank of American surgeons, and his death leaves a gap therein not easily filled.

